# ‘What kind of life is this?’ Diabetes related notions of wellbeing among adults in eastern Uganda and implications for mitigating future chronic disease risk

**DOI:** 10.1186/s12889-018-6249-0

**Published:** 2018-12-27

**Authors:** R. W. Mayega, E. Ekirapa, B. Kirunda, C. Nalwadda, J. Aweko, G. Tomson, C. G. Ostenson, J. Van Olmen, M. Daivadanam, J. Kiguli

**Affiliations:** 1 Department of Epidemiology and Biostatistics, Makerere University College of Health Sciences, School of Public Health, P.O. Box 7072, Kampala, Uganda; 2 Department of Health Policy, Planning and Management, Makerere University College of Health Sciences, School of Public Health, Kampala, Uganda; 3 Department of Community Health and Behavioural Sciences, Makerere University College of Health Sciences, School of Public Health, Kampala, Uganda; 4 Department of Public Health Sciences Karolinska Institutet, Stockholm, Sweden; 5 Department of Learning, Informatics, Management and Ethics (LIME), Karolinska Institutet, Stockholm, Sweden; 6 Institute of Tropical Medicine, Antwerp, Belgium; 7 Department of Food, Nutrition and Dietetics, Uppsala University, Uppsala, Sweden; 8 Swedish Institute for Global Health Transformation (SIGHT), Royal Swedish Academy of Sciences, Stockholm, Sweden

**Keywords:** Type 2 diabetes, Wellbeing, Notion, Overweight, Hypertension, Chronic disease

## Abstract

**Background:**

Effective prevention and care for type 2 diabetes requires that people link healthy behaviours to chronic disease-related wellbeing. This study explored how people perceive current and future wellbeing, so as to inform lifestyle education.

**Methods:**

Eight focus group discussions and 12 in-depth interviews were conducted in Iganga and Mayuge districts in rural Eastern Uganda among people aged 35–60 years in three risk categories (1) People with diabetes, (2) people at higher risk of diabetes (with hypertension or overweight) and (3) community members without diabetes.

**Results:**

People define wellbeing in three notions: 1) Physical health, 2) Socio-economic status and 3) Aspirational fulfilment. Most people hold the narrower view of wellbeing that focuses on absence of pain. Most overweight participants did not feel their condition as affecting their wellbeing. However, for several people with hypertension, the pains they describe indicate probable serious heart disease. Some people with diabetes expressed deep worry and loss of hope, saying that ‘thoughts are more bothersome than the illness’. Wellbeing among people with diabetes was described in two perspectives: Those who view diabetes as a ‘static’ condition think that they cannot attain wellbeing while those who view it as a ‘dynamic’ condition think that with consistent treatment and healthy lifestyles, they can be well. While many participants perceive future wellbeing as important, people without diabetes are less concerned about it than those with diabetes. Inadequate knowledge about diabetes, drug stock-outs in health facilities, unaffordable healthier food, and contradictory information were cited as barriers to future wellbeing in people with diabetes.

**Conclusions:**

To make type 2 diabetes prevention relevant to healthy people, health education messages should link current lifestyles to future wellbeing. Diabetes patients need counselling support, akin to that in HIV care, to address deep worry and loss of hope.

**Electronic supplementary material:**

The online version of this article (10.1186/s12889-018-6249-0) contains supplementary material, which is available to authorized users.

## Background

Because of the increasing burden of type 2 diabetes in low- and middle- income countries (LMICs) [1], a major shift in their health systems is necessary, to integrate prevention [2, 3]. Behavioural measures to prevent type 2 diabetes have been known for decades [4–6] and include: Healthy diets, physical activity, self-care and regular health check-ups [7, 8], and cessation of smoking and harmful alcohol intake [6]. Despite the available evidence however, behavioural interventions at the population level are largely inexistent in many LMICs [9].

Currently, the bulk of health education messages in sub-Saharan Africa are oriented towards acute infectious diseases [3]. Culturally-relevant interventions targeting chronic diseases require an understanding of how people perceive risk, and what motivates them to change behaviour [10, 11]. Such information is lacking for many contexts in sub-Saharan Africa [9]. The Value Expectancy Theory postulates that for behaviour change communication to be effective, it has to clarify the incentive for which individuals should adjust their behaviour [12]. The promise of wellbeing is one of the most authentic incentives for chronic disease related behaviour change [13]. However, it is not clear how communities in LMICs interpret current and future wellbeing in the context of their behaviour.

Uganda is undergoing the epidemiological transition to NCDs [14]. Prevalence of NCD risk factors like hypertension and overweight in some communities is substantial [15–17]. However, not only is Uganda’s health system largely oriented towards acute care [3, 18], but community knowledge about NCD risk factors is very low [19]. There is a paucity of evidence on how messages on prevention should be packaged to promote population level behaviour change. One such grey area is how healthy people perceive current and future wellbeing in relation to NCDs. For people who have already acquired chronic diseases like diabetes, it is possible to lead a relatively normal life [20, 21], similar to ‘positive living’ in HIV care. However, the extent to which they describe wellbeing in contexts like rural Uganda has not been assessed. At least one formative study has described perceptions about diabetes and the behaviours associated with it in Uganda [22]. That study showed that the recommended lifestyle adjustments are viewed by society as ‘sacrificing a good life’ [22]. However, that study fell short of describing how people define wellbeing.

The World Health Organization (WHO) describes wellbeing as composed of two domains, subjective and objective [23]. Taken synonymously with ‘quality of life’, the objective domain is assessed by standardized tools like the WHO Quality of Life Questionnaire. Huber and colleagues argue that WHO’s definition of health and wellbeing is insufficient in light of the increasing importance of chronic diseases and propose inclusion of ability to adapt and self-manage in the face of stresses [24]. Dooris et al. (2017) and Johnson and Acabchuk (2018) argue for a more inclusive definition that takes care of physical, biological and social aspects [13, 25]. In a review of nine wellbeing scales, Mc Dowell notes that the definitions of wellbeing remain open to considerable debate [26]. Subjective wellbeing is even more challenging because it is an individualised issue, implying that a cross-cultural definition is difficult to obtain [23]. In general it is not known how individuals in predominantly rural areas in sub-Saharan Africa view wellbeing in relation to chronic disease risk. Such knowledge would inform the development of health education messages that address subjective perceptions to promote healthy living in a context where NCDs are an emerging public health threat. The main objective of the present study therefore was to determine how adults with and without type 2 diabetes in Eastern Uganda perceive wellbeing and its implications for a healthy future.

## Methodology

### Study site and population

The study was conducted in Iganga and Mayuge districts in Eastern Uganda, which are the implementation sites for the project titled: “A people-centred approach through Self-Management and Reciprocal learning for the prevention and management of Type-2-Diabetes” (SMART2D). The districts are located approximately 120 km from Uganda’s capital. Iganga has an estimated population of 466,200 while Mayuge has 461,200. About 93% of their population resides in rural areas. Small scale agriculture is the main economic activity, but petty trading is common in the semi-urban areas. The study population were male and female adults aged 35–60 years living in the Iganga-Mayuge Health and Demographic Surveillance Site (IMHDSS) [27], an area that covers 65 villages. The HDSS conducts regular surveillance of vital statistics in an open cohort of 85,000 individuals.

#### Context in relation to lifestyle diseases

Iganga district, like Uganda in general is in the early stages of the epidemiological transition and communicable diseases still account for the bulk of morbidity. Local health systems and health promotion activities are still largely centered on acute conditions [28] . There are no formal health promotion programs targeting lifestyle diseases either in health centres or at community level. As a result, awareness about lifestyle diseases both in the population and among health care workers is low [17]. While there are community health workers who support health promotion, their awareness about lifestyle diseases is very low.

#### SMART2D

This study was part of the formative phase of SMART2D, an implementation research project aimed at strengthening type 2 diabetes (T2DM) prevention and care by translating available knowledge into smart interlinked interventions adapted to different settings. The specific objective of SMART2D is to formulate and evaluate a contextually appropriate self-management approach for prevention and control of T2DM through facility and community components. The project targets adults at high-risk for or diagnosed with T2DM in three settings: a rural area in a low-income country (Uganda), an urban township in a middle-income country (South Africa) and a vulnerable immigrant community in a high-income country (Sweden). The project intends to translate the research findings into inputs for national guidelines and policies. This study’s findings fed into development of intervention strategies for the SMART2D trial [29].

### Study design

The design of the study was a crossectional qualitative study employing two approaches: focus group discussions (FGDs) and in-depth interviews (IDIs). Study participants were purposively selected from three categories of people in the study area: (1) People with diabetes aged 35 to 60 years, (2) people at high risk of diabetes (i.e. with hypertension or overweight) aged 35 to 60 years, and (3) people without diabetes aged 35 to 60 years from the community. FGDs were conducted in all three categories while IDIs interviews were conducted in two of the categories, excluding the category of community members without diabetes (See Table 1). The reason why IDIs were excluded from the category of community members was that IDIs go to the depths of individual experiences yet the general community members did not have reasonable experience with NCD related risk factors or diabetes because of the generally low awareness about NCDs and diabetes. The FGD and IDI approaches allowed participants to describe their feelings and perceptions on the issues for discussion [30]. The three risk categories made it possible to compare views from people with different risk profiles.

**Table 1:** Sample distribution for the data collection activities

Site	Participant category	Methods and respondents
Iganga-Mayuge DSS (randomly selected villages, 2 hospitals and other health facilities)	Diabetes patients and people at higher risk of diabetes, stratified by gender	8 IDIs 2 females with diabetes 2 males with diabetics, 2 males with risk factors 2 females with risk factors
	Diabetes patients, people at higher risk of diabetes, people without diabetes from the community stratified by gender	12 FGDs 2 for females with diabetes 2 for males with diabetes 2 for males with risk factors 2 for females with risk factors 2 for males from the general community 2 for females from the general community

### Sample selection

Twelve FGDs and 8 in-depth interviews were conducted. This number was reached by continuing the interviews until saturation of ideas was realized in the three participant categories targeted (people with diabetes, people at high risk for diabetes, and community members without diabetes). The FGDs were conducted in all three risk categories. The three categories were also stratified for sex resulting in six categories. For each of the six categories, two FGDs were conducted to ensure availability of two sets of opinions. The FGDs comprised an average of 8 people each (range of 6 to 10 participants). The two risk categories in which in-depth interviews were held (people with diabetes and people at high risk for diabetes) were also stratified by sex, and two IDIs conducted in each sub-category.

The Clinical Officers (a cadre equivalent to assistant doctors) working at the outpatient departments in the study area identified the people with type 2 diabetes and the people at higher risk for type 2 diabetes. People with diabetes were identified from the diabetes or outpatient clinic at Iganga Hospital, from where those who fulfilled the inclusion criteria were consecutively selected and allocated purposively to the FGDs or to the IDIs. Those with hypertension or overweight were identified consecutively from adults seen in at Iganga hospital and two health centres level IV outpatient clinics for minor ailments. The third category of participants (i.e. community members without diabetes) were identified from the communities in the DSS. To get a representative sample of these community members, the study villages were stratified into urban-rural from which three urban and 10 rural villages were randomly selected. ‘Community scouts’, a cadre of community level research assistants who guide DSS research assistants in the routine data collection rounds, visited the households consecutively recruiting willing participants until the required numbers were realized. In total, 106 people participated, 64 of whom were females (60.4%). The overall median age was 49 years. Majority of the participants resided in Iganga district 80/106 (75.5%). Most of the participants were married 79/106 (74.5%).

To be included in the study, people with diabetes had to be aged atleast 18 years, and to have a diagnosis of diabetes confirmed atleast 6 months prior to the study. People with risk factors had to be aged atleast 18 years, who had either a diagnosis of hypertension confirmed at least 6 months before the study or were assessed to have obesity at the time of the study (defined as a Body Mass Index greater than 30). Community members had be aged atleast 18 years and to have been resident for at least 6 months in the 13 selected villages for the study. Only participants with sound mental health status and who were not severely ill to the extent of requiring hospitalization were included.

### Data collection

The interviews and FGDs were arranged at a time and place that was convenient for the participants. Data were collected by three investigators and six research assistants conversant with the local district context, language and with experience in conducting qualitative studies. They formed three data collection teams, each comprising one of the investigators and two research assistants. All interviews were tape recorded. The interviews were led by the principle investigator (RWM) and co-investigators (EE and CN) while one of the research assistants managed the recording process, and the second research assistant took manual notes on emerging issues.

The data collection guides used for this study were designed anew by the investigators with a view to exploring perceptions on three thematic issues: (1) Lay notions and perceptions of wellbeing; (2) Attitudes towards behaviour change, and (3) Possible actions, enablers and barriers to current and future wellbeing. The locus of the tools was to explore participants’ own subjective views of wellbeing rather than perspectives from objective tools. The dimensions and questions used in the guide were derived from an open brain-storming session by a team of SMART2D investigators. To enhance content validity of the tools, the FGD guide was reviewed by a medical anthropologist (JK), public health specialists (RWM, BK, and MD), a qualitative methods specialist (CN), and a health systems specialist (EE), who then agreed on the final interview items. The guide was translated to the local language (Lusoga) and back-translated to English for accuracy. A copy of the questions asked is provided as a supplement (Additional file 1).

### Data management and analysis

FGD recordings were labelled and stored. They were then transcribed and translated into English by three experienced research assistants fluent in both languages. The authors namely: RWM (a public health specialist), JK (an anthropologist), EE (health systems), BK (diet and physical activity), CN (qualitative methods), MD (nutrition and health systems), read the transcripts, and discussed emerging themes. Thereafter three investigators (RWM, JK, and CN) developed a codebook. They did this by selecting three transcripts, re-reading them, assigning meaning units to each response and codes to each meaning unit while also taking notes on emerging sub-themes. They then met, combined their descriptive codes and discussed them to come up with a unified code book. Data was entered in Atlas Ti® software version 7.0 to help in code assignment and axial coding.

Qualitative data was analyzed through thematic analysis, to identify recurrent themes and subthemes by systematically categorizing the transcript codes [31]. This approach is suitable for analysis of qualitative data in multi-disciplinary health research [32]. An team approach to identification of higher level themes was used, applying inductive reasoning [33]. Data analysis started one month after the data collection upon completion of the data transcription and translation and continued for six months. Cognizant that FGDs and IDIs are likely to produce different outcomes in terms of social constructions vs. individual perceptions, the analytical approach initially focused on individual perceptions contributed by different discussants, to enable triangulation of emerging themes across methods. The FGDs then added a perspective on what emerged as consensus patterns in the groups and across groups.

### Validity and trustworthiness

A number of methodological approaches were used to promote validity. The multi-disciplinarity of the study team brought different perspectives into the study. Follow-up questions and probes were used to validate responses during the interviews. Three coders developed the analysis code-book to enhance its reliability. The face-to-face interaction in a discussion mode with the participants promoted trustworthiness. Trustworthiness of findings was also enhanced through consistency checks framed as follow up questions and assertion probes, triangulation of the findings from the two methods (FGDs and IDIs) and conducting atleast two FGDs in each of the three participant categories [34].

## Results

Findings are presented under four emerging themes: 1) How individuals define wellbeing; 2) Relationship of wellbeing to having diabetes; 3) Concern about future wellbeing; and 4) Actions needed to promote future wellbeing. These findings are summarised in Table 2.

**Table 2:** Summary of results, emerging sub-themes and themes

Meaning unit	Condensed meaning unit	Category*			Sub-theme	Theme
		COM	PWD	PAR		
*“Being well means you don’t have illnesses…. no source of pain in the whole body” (FGD, Community members, Male)* *“If you don’t have money, you can’t afford….necessities; therefore you cannot feel well” (Person with Diabetes, Male)* *“(When) There is nothing disturbing my mind. I have no fear or worries…my life is at peace” (FGD community members, Female)*	• Wellbeing is defined in three main notions of increasing complexity: 1) Physical health/pain, 2) socio-economic status and 3) aspirational fulfilment.	√	√	√	Notions attached to wellbeing	How individuals define wellbeing
*“Pressure has affected me a lot. Small things frighten me. I get headache….. I also get stomach pain, chest pain and others. When I walk a small distance, I get tired, I get palpitations.” (FGD People at Risk, Female)*	• Among participants at higher risk of diabetes:				Some risk factors affect subjective wellbeing; others do not	
	o The majority of overweight participants feel weight does not affect their wellbeing			**√**		
	o Those with hypertension report pains indicative of potentially severe disease			**√**		
*“With HIV you can feel well. Not with diabetes! Even if you are happy now, there is pain underneath because sickness can happen anytime. (KI, Male with diabetes).”* *“Diabetes doesn’t mix with parties. A little alcohol and you collapse…You have to watch as your friends enjoy. What kind of life is this?” (FGD, Diabetes, Female).* *“If you follow the guidelines, your glucose will balance… people will doubt whether you have diabetes.” (FGD, Person with diabetes, Male)* *“We are happy if we get all the medicines. We can’t afford them…we don’t bother buying them” (FGD, Person with diabetes, Male)*	• Participants attach two sub-notions to being well when one has diabetes:				Notions attached to well-being with diabetes	Relationship of wellbeing to having diabetes
	o Diabetes as a ‘static state’: Regardless of treatment, a person with diabetes cannot be well	**√**	**√**	**√**		
	o Diabetes as a ‘dynamic state’: People with diabetes get long periods in which they feel well		**√**			
	• However, wellbeing with diabetes is conditional to assurance of continuity of services and availability of medicines		**√**			
*“You go back home with deep thoughts; Because you cannot get healed, you feel powerless. Thoughts are more painful than the disease.” (FGD, Person with diabetes, Female)*	• Many people with diabetes seemed deeply bothered by thoughts, which severely impacted on their current and future wellbeing.		**√**		Psychosocial well-being a key factor	
*“It’s important to be well so you can plan for your children….that the plans I have today are realized in the future” (FGD, PAR Male)* *“Diabetes is a normal disease but it is not like malaria which goes away. If you prepare your body to prevent future illnesses, future life will be sweeter” (FGD, Person with Diabetes, Female)*	• People without diabetes: motivation for future wellbeing is from ability to fulfil future plans	**√**		**√**	Differing motivators for well-being	Concern about future wellbeing
	• People with diabetes: Future health is central to future wellbeing.		**√**			
*“I have high hopes…am not going to die. I have lived with diabetes for 10 years. When I had just contracted it, I felt so bad. But since I started coming to the hospital, I am fine” (FGD, Person with diabetes, Male)* *“Although I am young, my hope for a healthy future reduced; .nothing to look forward to…A friend who had diabetes for only a few months passed away; I lost hope” (FGD, People with Diabetes, Female)*	• Many people with diabetes were optimistic about the future.		**√**		Optimism about the future differs	
	• However, older people/people who have lived with diabetes longer had a more positive attitude		**√**			
	• On the other hand, several younger people with diabetes had lost hope in the future.		**√**			
*“We have to work for our children. I am planting trees, coffee; constructing rentals. Even if I become weak, these can pay school fees and feed me.” (FGD Community members)* *“I follow the regulations. I cut the sugar. I eat lot of greens. I eat some starchy foods. Plus eating in time. When hungry, I eat something small. I don’t walk like others - I walk fast!” (FGD People with diabetes)*	• Regarding actions for future wellbeing:				Actions for future wellbeing differ	Actions to promote future well being
	o People without diabetes tended to suggest investments for future economic wellbeing.	**√**		**√**		
	o People with diabetes on the other hand cited adherence to medication and healthy lifestyles		**√**			
*“We have not been sensitized enough about the way we should behave” (FGD Community Member)* *“Health workers told us: Don’t eat sweet potatoes, sweet bananas and mangos. But when we went to Mulago, they told us that fruits are good for the body but don’t eat to get satisfied.” (FGD People with diabetes)*	• Barriers to future wellbeing with diabetes: Lack of knowledge, stockouts of drugs in health facilities, inability to afford healthier food, and contradictory messages from health workers as.		**√**		Barriers to future wellbeing	

* Category: *COM* = Community; *PWR* = People with Risk Factors; *PWD* = People with Type 2 diabetes

### How individuals define wellbeing

#### Notions attached to wellbeing

**Fig. 1:**
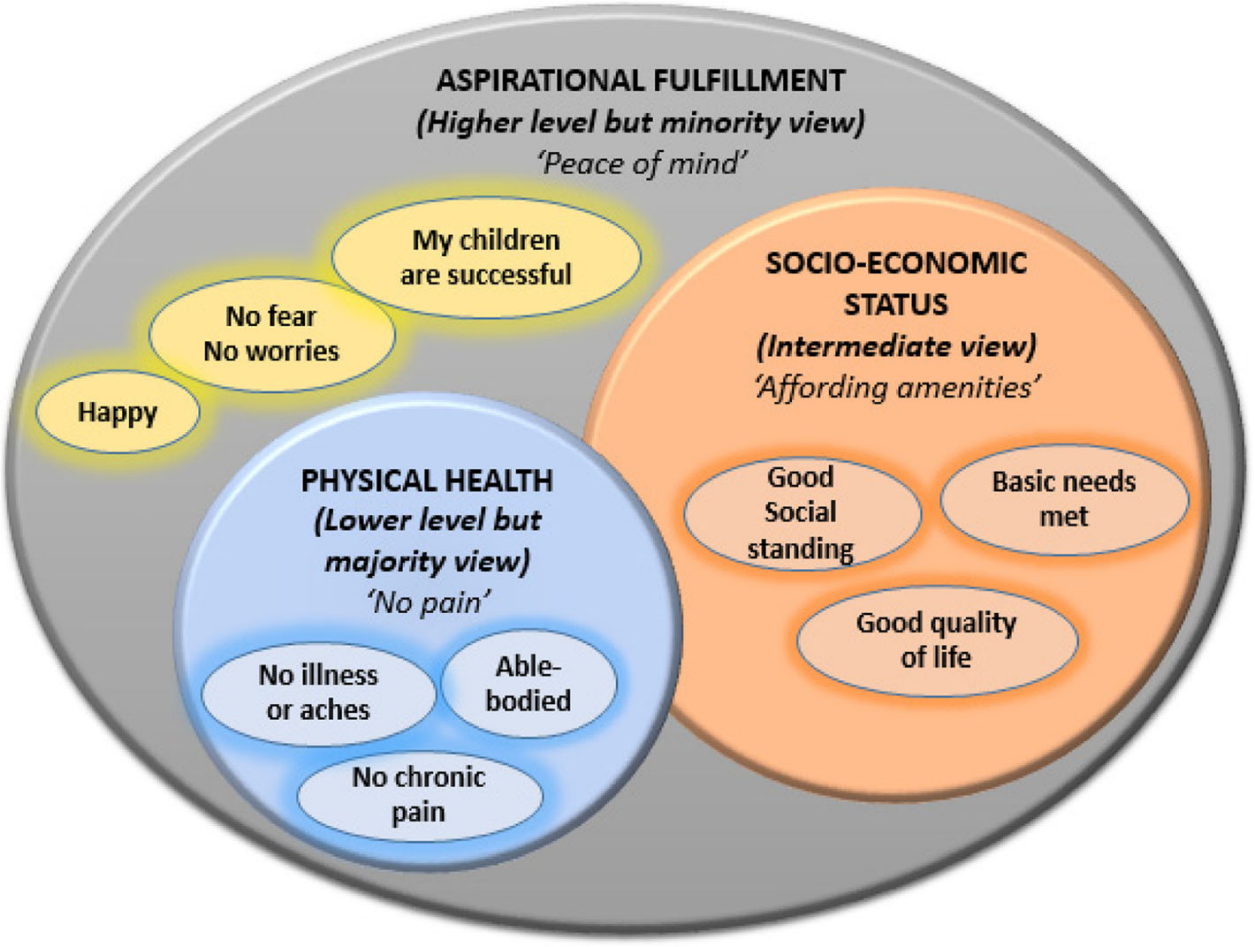
Notions attached to wellbeing

Wellbeing was defined in three main notions, showing increasing complexity: 1) Physical health/pain, 2) socio-economic status and 3) aspirational fulfilment (Fig. 1). In the first notion, held by a majority of participants (7/12 FGDs; 5/8 KIs), participants view wellbeing narrowly as the absence of illness, pain or discomfort.

*“Being well means you don’t have illness including invisible illnesses. You have no source of pain in the whole body” (FGD, Community members, Male).*

Some discussants include long term illnesses in this notion *(*e.g.: *‘I am well when upon being checked, I have no anaemia, no high-blood pressure, or no diabetes*’), while others include being physically able-bodied or unencumbered:

*“Wellbeing is when I have nothing affecting my body parts; your muscles are active. You can do any work that requires energy without difficulty. If you want to walk on foot you can do so easily” (FGD Community members, Female).*

In the second notion (3/12 FGDs; 2/8 IDIs), participants associate wellbeing to socio-economic status, which includes having sufficient income to afford basic necessities, good food (which they characterize as *‘tasty or savoury food’*), amenities that foster a good quality of life, and having a good social standing.

*“If you don’t have money, you can’t afford necessities and therefore you cannot feel well. With money, you can feed your family and pay school fees; you eat nice tasty food; and you are ‘somebody in society’” (IDI, Person with Diabetes, Male).*

In the third notion, which was the least prevalent (2/12 FGDs; 1/8 IDIs), participants refer to wellbeing as a higher status of aspirational fulfilment beyond health and socio-economic status and includes *‘having a life which others aspire to have’*, *‘peace of mind’ and ‘success of dependants’*. To people in this category, wellbeing is synonymous with happiness.

*“There is nothing disturbing my mind. I have no worries about poverty, feeding, or illness; when my life is at peace; my children have studied and are successful; they are able to live gainfully” (FGD community members, Female).*

#### Diabetes related risk factors and wellbeing

Among participants at higher risk of diabetes, most overweight participants said their weight does not affect their wellbeing but those with hypertension said that having hypertension affects them substantially. The pains that some participants associate with ‘hypertension episodes’ might me symptoms of severe undetected disease of the heart (chest and abdominal pain), the nervous system (headaches, paralysis, blurring of vision) and the musculo-skeletal system (joint, leg, muscle pain).

*“Pressure has affected me a lot. Small things frighten me. When someone bangs the door my pressure shoots up. I get headache - like there is a charcoal stove in my head. I also get stomach pain, chest pain and others. When I walk a small distance, I get tired, I get palpitations.” (FGD People at Risk, Female).*

*“Sometimes you are seated there and you feel it (the pressure) rising; if it (the pressure) is for the eyes, you feel the eyes ‘dying’, the brain spinning, the ears blocked. The other type is for sweating: You feel you have no peace; your heart beats a lot, you start shaking and sweating.” (FGD, People at Risk, Male).*

### Relationship of wellbeing to having diabetes

#### Notions attached to wellbeing with diabetes

Participants attach two sub-notions to being well when one has diabetes: The first notion, carried by most participants without diabetes (7/8 FGDs) and some with diabetes (2/4 FGDs) is that regardless of treatment, a person with diabetes cannot be well because ill-health is always lurking. They view diabetes as a ‘static state’ in which a person is always or will always fall ill intermittently (*‘a few good and many bad days’*). They cite uncertainty of when ill-health will come, bother from symptoms, food restrictions, inconvenience to family, and endless medication, and not being able to enjoy some life activities as making wellbeing impossible. Some of them feel that their quality of life is worse than that of people with HIV.

*‘With HIV you can feel well. Not with diabetes! Even if you are happy now, there is pain underneath because sickness can happen anytime. (IDI, Male with diabetes).*

*“Diabetes doesn’t mix with parties. If there is a disco and you dance, that may spell trouble. A little alcohol and you collapse. If you join others in celebrating, headache starts. You’ll be rushed to Nakavule HCIV for a drip. You have to sit and watch as your friends enjoy. What kind of life is this?” (FGD, Diabetes, Female).*

The second category of participants (mostly people with diabetes) (all 3 FGDs) are more optimistic, citing that a person with diabetes can be well for long-periods and that even people without diabetes get ‘bad days’ anyway (‘Diabetes as a dynamic state’). They argue that consistent treatment, adherence and healthy lifestyles lead to control of blood sugar and a reduction in worrisome thoughts which leads to wellbeing, but this is ‘hard work’.

*“If you follow the guidelines and treat yourself well, your glucose levels balance in the body; people will doubt whether you have diabetes. Your diabetes is low.” (FGD, Person with diabetes, Male).*

*“When you feel some improvement, don’t be tempted to stop treatment. When I went home, I was like ‘these people are lying, I feel better; my diabetes is healed’. Shortly after, the problems started again.” (IDI, Person with diabetes, Female).*

However, people with diabetes said that compliance with treatment alone is not sufficient for wellbeing. They were continually concerned about continuity of services, especially availability of medicines in health centres.

*“We are happy if we get all the medicines. We can’t afford them. If there are no medicines, the only treatment we can undertake is to avoid certain foods-but we don’t bother buying the medicines. Sometimes health workers are very rude and not ready to listen!” (FGD, Person with diabetes, Male).*

This notional category included a sub-category of participants (all of whom were without diabetes) who view chronic disease as not one continuous illness but each episode as an acute illness on its own (‘diabetes as a series of illness episodes’).

#### Psychosocial well-being a key factor

Many people with diabetes seemed deeply bothered by thoughts, which severely impacted on their current and future wellbeing. Bother from recurrent illness, endless treatment and complications was the main cause of thoughts. To some people with diabetes, thoughts are more bothersome than the physical illness.

*“You go back home with deep thoughts; you haven’t gotten drugs; you don’t have money to feed your family or pay school fees; they tell you not to eat almost everything. It is difficult even on our caretakers; they put pressure on you. Because you cannot get healed, you feel powerless. You count yourself among the dead. Thoughts are more painful than the disease.” (FGD, Person with diabetes, Female).*

### Concern about future wellbeing

#### Whether future well-being is important

Many participants, regardless of diabetes status, perceive future wellbeing as important to their current life. However for most people without diabetes, their motivation for future wellbeing was less from anticipated health benefits but more about ability to fulfil future plans and look after their dependants.

*“It’s important to be well because you can plan for your children; if am weak, I cannot be useful to the country and my children. I also want to see that the plans I have today are realized in the future” (IDI, PAR Male).*

On the other hand, people with diabetes tended to mention future health as central to their future wellbeing, and that despite their incurable condition, they can have long lives.

*“I take diabetes to be a normal disease but it is not like malaria which goes away. It is not going to kill me now. If you prepare your body to prevent future illnesses, future life will be sweeter than present life” (FGD, Person with Diabetes, Female).*

#### Optimism about the future

Many people with diabetes were optimistic about their future. However, people who have lived with diabetes longer had a more positive attitude than those who had been diagnosed recently.

*“I have high hopes because am not going to die tomorrow. I have lived with diabetes for 10 years. When I had just contracted it, I felt so bad was so thin and I did not have hope. I would be admitted every week, sometimes in a coma. But since I started coming to the hospital, I am fine. If I don’t tell someone that I have diabetes, one cannot know” (FGD, Person with diabetes, Male).*

On the other hand, several younger people with diabetes, said they had lost hope in the future. Bother from recurrent illness and complications, dietary restrictions, being on insulin, and loss of hope, were cited as the main factors behind this.

*“Although I am young, my hope for a healthy future reduced. There is nothing to look forward to. The way diabetes affects us differs. It spares those who have spent 30 years with it and is killing the younger ones. When a friend who had diabetes for only a few months passed away, I lost hope” (FGD, People with Diabetes, Female).*

*“Some people, mainly those on injections, give up hope. I have seen several of them because we visit fellow patients. They say ‘let me die’. They resume eating oily food, sugar, and even stop the injections” (FGD, People with Diabetes, Male).*

Loss of sexual performance was particularly concerning to male diabetes sufferers:

*“If I have lost manpower, why should I continue living? Our young wives can’t be patient and we need joint counselling. Instead of being supportive, she laughs while telling others your secrets: ‘my husband is like this’ and you get to know. Then they run to another man and you are ruined” (FGD, People with Diabetes, Male).*

### Actions that need to be taken to promote future well being

#### Actions for future wellbeing

People without diabetes differed from those with diabetes regarding actions that need to be taken to promote future wellbeing. Those without diabetes tended to suggest investments for economic wellbeing and educating children.

*“We have to work for our children. I am planting trees and coffee and constructing rental rooms. Even if I become weak, these investments can pay school fees and feed me.” (FGD Community members).*

People with diabetes on the other hand cited adherence to medication and healthy lifestyles as actions needed to promote future wellbeing:

*“Although I have diabetes, I have avoided other diseases like pressure. I follow the regulations. I reduced sugar and I eat lot of greens. I eat starchy foods except cassava which has too much starch. Plus eating in time. When I feel hungry, I eat something small. I don’t walk like people here do - I walk fast!” (FGD People with diabetes).*

*“When I woke up, my blood sugar was high. I recorded it here: 16.4 at 7:47 am. Then I rode my bicycle 12 km to town. Since I returned, my blood sugar is 6. I was urinating frequently but this has stopped. (FGD People with diabetes, Male).”*

While people with diabetes constantly try to search for information regarding healthy lifestyles, those without diabetes do not normally do so, including those at higher risk of diabetes. Many participants link bitter herbal supplements to future wellbeing.

*“For my wellbeing in future, I don’t fail to eat a bitter thing every day like Aloe Vera, bitter lemon and bitter leaves of the ‘Jambula’ tree.”*

#### Barriers to future wellbeing

Participants cited lack of knowledge, stockouts of drugs in health facilities, inability to afford healthier food, and contradictory messages from health workers as barriers to future wellbeing. They said that one health worker might tell them to implement a given behaviour (e.g. related to diet), and another one tells them that it is unhealthy. They also said that the youth’s mind-set does not tend to think about the future.

*“We have not been sensitized enough about the way we should behave. If there was a special way in which we can be educated about how we are supposed to eat and behave. And if everybody could be tested” (FGD Community Member).*

*“Some health workers told us: Don’t eat sweet potatoes, sweet bananas and mangos. But when we went to Mulago, they told us that fruits are good for the body but don’t eat to get satisfied. They said: for jack fruit, eat about two pieces; for sweet bananas, eat one large one or two small ones.” (FGD People with diabetes).*

*“In the youthful ages, there is no plan. Because one feels the energy, it does not even occur to them to live healthy. They drink hard alcohol that weakens the body. The youthful mind thinks and acts on the moment” (IDI, Person at Risk).*

## Discussion

This study brings to light the notions that people with and without diabetes attach to current and future wellbeing and their gradient of complexity. It explores how people at higher risk for diabetes perceive their wellbeing. It also explores whether people think that it is possible for a person with diabetes to be well, in light of the emerging calls for a new definition of health that includes ability to adapt and self-manage due to the increasing view of chronic diseases as life conditions [24]. We highlight how people perceive future well-being, and perceived barriers to acting towards it, showing that in all these aspects, people with diabetes differ from those without diabetes.

We found that most people have a narrow view of wellbeing that focuses on absence of pain and access to basic needs. This thinking aligns with Maslow’s Hierarchy [35], showing that our study population mainly focuses on more basic and intermediate needs, the type that Steptoe and colleagues characterize as ‘hedonistic wellbeing’ [36]. The ‘sense of purpose’ notion labelled by Steptoe and colleagues as ‘evaluative wellbeing’ [36] is only reflected in our participants when they express the aspiration to ensure that their off-spring are able to succeed in the future. Dooris et al. highlight the importance of looking at wellbeing beyond health and happiness to include effective functioning, sense of purpose, and flourishing (i.e. holistic wellbeing) [13]. The finding that overweight people do not think that their weight affects their wellbeing is rooted in a disease paradigm common in Africa in which ill health is associated with acute symptoms [14, 37]. In fact, an earlier study in this setting showed that being overweight was associated with success [22]. Chronic diseases are often preceded by long periods in which people feel well but may have undetected signs [18].

This study also finds that some pains that people with hypertension have might be symptoms of potentially serious heart disease. Hypertension is usually a symptomless risk factor, but if people feel symptoms, they might actually have undetected cardiovascular diseases like coronary heart disease, arrhythmias or hypertension related cardiomyopathies. A systematic review of Ischaemic Heart Disease in Africa notes that there are high rates of non-specific ECG changes suggestive of myocardial ischaemia in up to 10% of asymptomatic African men and 20% of women over the age of 40 years [38].

Our findings on whether a person with a serious chronic condition like diabetes can be well were equivocal: Some participants think that people with diabetes cannot be well while others think the opposite. Other studies in Africa have also shown diabetes patients to view their condition as worse than HIV [39, 40]. A study that compared diabetes and HIV care environments in Tanzania draws clear parallels in quality and maturity of HIV services compared to diabetes services [41]. Participants in our study cite restrictions from ‘almost everything’ as severely impacting on their quality of life. Some people with diabetes in our study think that achieving wellbeing is conditional to having consistent quality treatment including free medicines, adherence, and healthy lifestyles, which they label as ‘hard work’. Similar perceptions were observed in the Kilimanjaro Diabetes project [42]. In another study, diabetes patients were explicit in stating ‘it is the medicines that keep us alive’ [43]. Studies in South Africa and Tanzania highlight a high unmet need for diabetes care in Africa [44, 45]. Our study affirms that gaps in continuity of services in low income countries impact severely on perceived wellbeing for people with diabetes.

While for people with diabetes, actions for future wellbeing are motivated by a healthy future, those without diabetes are preoccupied by future socio-economic wellbeing. This trend aligns with the Value Expectancy Theory which views behavioural choices as a function of expectations and the value attached to an outcome [12]. The theory expounds that when more than one behaviour is possible, the behaviour chosen will be the one with the largest combination of expected success and value for a particular individual’s experience [12]. As an example, people with diabetes tended to search for information about healthy lifestyles while those without diabetes did not. These differences in motivation for future wellbeing actions between people with and without diabetes require targeted approaches to promotion of healthy lifestyles in the two categories of people.

Barriers to taking actions for future wellbeing for people without diabetes (including those with risk factors) are rooted in their lack of awareness about diabetes prevention. On the other hand, healthy choices among people with diabetes are curtailed by inaccess: Unstandardized information, unaffordability of healthy food, and interrupted treatment due to drug stockouts. Murphy et al. found similar observations among diabetes patients in South Africa [46]. Adequate information is cited as a pre-requisite for achieving what Ellis et al. describe as ‘the good self-manager’ [47]. Our study finds that young people without diabetes are not preoccupied with healthy living. Externalization of diabetes risk has been demonstrated in other studies among young people [48, 49]. Conversely, younger people with type 2 diabetes seem to be struggling to cope with the disease.

Some participants with diabetes describe feelings of powerlessness, extreme worry and loss of hope, which they ascribe to uncertainty, complications, dietary restrictions, endless medication, and loss of sexual ability. In a study on illness experiences in Tanzania, many people with diabetes were continually worried about unpredictable ailments and loss of consciousness, memory, libido, and function [50]. These characteristics align with the Salutogenic Theory proposed by Antonovsky in 1979 to explain patients’ ability to cope with stress [51]. The central tenet of this theory, the concept of ‘sense of coherence’ is suggested to comprise three drivers of coping and adaptation to chronic illness: comprehensibility, manageability, and meaningfulness [51]. Mendenhall and colleagues observe that anxiety and depression among diabetes patients is understudied yet high [52]. The findings indicate urgent need for structured counselling services for people with type 2 diabetes.

## Conclusions and implications for policy

Regardless of diabetes status, people have a narrow view of wellbeing. Preventive efforts for diabetes should make the message of future health and wellbeing relevant for people who feel well now and should promote a holistic view of current and future wellbeing as an incentive for behaviour change. Further studies to investigate possible undetected heart disease among people with hypertension are needed. Health educators should portray diabetes as chronic condition in which people can be well for long periods of time if they adhere to treatment. To address severe psychosocial distress, counselling services should be integrated into the basic package for diabetes care, drawing lessons from HIV care. Services improvement to ensure continuity of diabetes care should be emphasized. Comprehensive population-based health promotion programs are necessary to create critical awareness about the link between current behaviour and future health so as to stem the rapid increase in behavioural risk factors in low income countries, in line with the 2030 Agenda and Universal Health Coverage. Researchers assessing wellbeing as an outcome of health interventions in this and related contexts should include the three notions identified in this study in their measurements.

Findings from this assessment will inform the development of a contextually relevant communication guide for increasing risk awareness and perceptions about healthy lifestyles in similar contexts in Uganda. The study also provides a formative basis for designing and implementing a study to quantify the extent of undetected hypertensive heart disease, stroke and transient ischaemic attacks in contexts such as Uganda. Lastly, it provides a strong basis for advocacy to improve the quality of diabetes care, including continuity of care, availability of medicines, patient education and establishing robust counselling services.

## Methodological considerations

The main methodological limitations of this study included not stratifying the respondent categories for other risk factor groups (like tobacco smokers and heavy alcohol takers), not anchoring the study in a quality of life theoretical framework and transferability. The interviews occurred in a context where awareness about chronic diseases was low, hence the higher focus on a narrower definition of wellbeing. Regarding risk factor groups, overweight and hypertension are the most prevalent risk factors for diabetes in this context. A theoretical framework was not needed since the study focused on the subjective aspect of wellbeing. The transferability of our findings applies to the socio-cultural context of predominantly rural communities in sub-Saharan Africa with low knowledge about chronic diseases and higher awareness about acute conditions. Consecutive recruitment of participants in the three study categories is acknowledged as a limitation that could likely have caused a selection bias. Interpretation of results therefore focused less on generalizability and more on transferability.

## Additional file

Additional file 1: Focus group discussion Guide. (DOCX 13 kb)

## Abbreviations

DSS: Demographic surveillance site
FGD: Focus Group Discussion
HDSS: Health and Demographic Surveillance Site
HIV: Human Immuno-deficiency Virus
IDI: In-depth Interview
IMHDSS: Iganga – Mayuge Health and Demographic Surveillance Site
LMIC: Low- and Middle- Income Country
NCD: Non-Communicable Disease
PAR: People at Risk
SMART2D: “A people-centred approach through Self-Management and Reciprocal learning for the prevention and management of Type-2-Diabetes”
T2DM: Type 2 Diabetes Mellitus
WHO: World Health Organization
