# Supplementary material for: ‘What kind of life is this?’ Diabetes related notions of wellbeing among adults in eastern Uganda and implications for mitigating future chronic disease risk
Source: BMC Public Health. 2018 Dec 27;18:1409. doi: 10.1186/s12889-018-6249-0 (PMC6307159; doi:10.1186/s12889-018-6249-0)
Supplement: Supplementary file 1 — Focus group discussion Guide. (DOCX 13 kb) [file 12889_2018_6249_MOESM1_ESM.docx]

**Focus group discussion Guide**

**Extract to assess notions of wellbeing**

1. **Notions of well being**

1.1 What do you consider as ‘well-being’ in terms of health?

Follow up: How would you describe a person who is ‘well’ health wise?

Probe: For physical well-being, well-being in terms of feelings and thoughts; Do you think that being happy is the same as being well?

1.2 Do you think that future well-being matters in a person’s life? Explain why or why not?

Follow up: Do you think that the way you lead your life today may affect your future wellbeing? If so how?

Probe: For positive and negative actions and impacts

1.3 Have you ever thought about your own future well-being? Please explain more

Follow up: Have you ever thought about doing somethings now so that your future health is good? Can you explain?

Probe: Have you done anything in the last 3 years with the aim of improving your wellbeing in the future? Can you explain?

1.4 Do you think that a person who appears perfectly well physically can be sick and unwell with regard to what you do not see? Explain why or why not.

Probe: Can people with hypertension but no diabetes be well (or for people with hypertension: Knowing that you have hypertension, does your condition affect you in any ways?)

Explore the same question for obesity: Can people with obesity but no diabetes be well (or for people with obesity: Knowing that you have hypertension, does your condition affect you in any ways?)

1.5 Do you think it is possible for a person with diabetes to have ‘wellbeing’ the way you have described it? Explain why or why not

Follow-up: What can a person with diabetes do to have a life as close as possible to the well-being you have described?

Probe: What are the challenges to achieving well-being among people with diabetes?
